# Interstitial lung disease in a patient treated with oxaliplatin, 5-fluorouracil and leucovorin (FOLFOX) for metastatic colorectal cancer

**DOI:** 10.2478/v10019-012-0006-2

**Published:** 2012-11-09

**Authors:** Liam M Hannan, Jaclyn Yoong, Geoffrey Chong, Christine F McDonald

**Affiliations:** 1 Department of Respiratory and Sleep Medicine, Austin Health, Heidelberg, Victoria, Australia; 2 Ludwig Oncology Unit, Austin Health, Heidelberg, Victoria, Australia; 3 Institute for Breathing and Sleep, Austin Hospital, Heidelberg, Victoria, Australia

**Keywords:** oxaliplatin, interstitial lung disease, diffuse alveolar damage

## Abstract

**Background:**

Oxaliplatin in combination with 5-fluorouracil (5-FU) and leucovorin (FOLFOX) is a common chemotherapeutic regimen for advanced colorectal cancer. Here, we present a case of interstitial lung disease associated with FOLFOX therapy.

**Case report:**

A 74-year-old man with a history of metastatic colorectal cancer was admitted with a four week history of progressive dyspnoea and evidence of severe respiratory failure. He had recently completed six cycles of FOLFOX chemotherapy in the months prior to presentation. Investigations did not reveal convincing evidence of infection or pulmonary embolism. CT chest demonstrated widespread pulmonary infiltrates and interlobular septal thickening. The patient was commenced on both broad spectrum antibiotic therapy and high dose corticosteroid treatment however his respiratory failure continued to progress. The patient died four days after admission due to progressive respiratory failure. Subsequent post-mortem examination demonstrated evidence of diffuse alveolar damage without evidence of tumour infiltration, infection or pulmonary embolism.

**Conclusions:**

Although infrequent, pulmonary toxicity can occur in association with FOLFOX therapy. Cessation of therapy and prompt initiation of corticosteroids may improve outcomes.

## Introduction

Oxaliplatin in combination with 5-fluorouracil (5-FU) and leucovorin (FOLFOX) is an effective chemotherapeutic regimen for advanced colorectal cancer, improving survival with acceptable tolerability and side effect profile.^1^–^3^ Common side effects from this regimen include neutropenia, sensory neuropathy and gastrointestinal symptoms.^1^,^2^ There have been infrequent reports of pulmonary toxicity from this regimen.^4^–^8^ Here we present a case of interstitial lung disease associated with FOLFOX therapy.

## Case report

**FIGURE 1.**
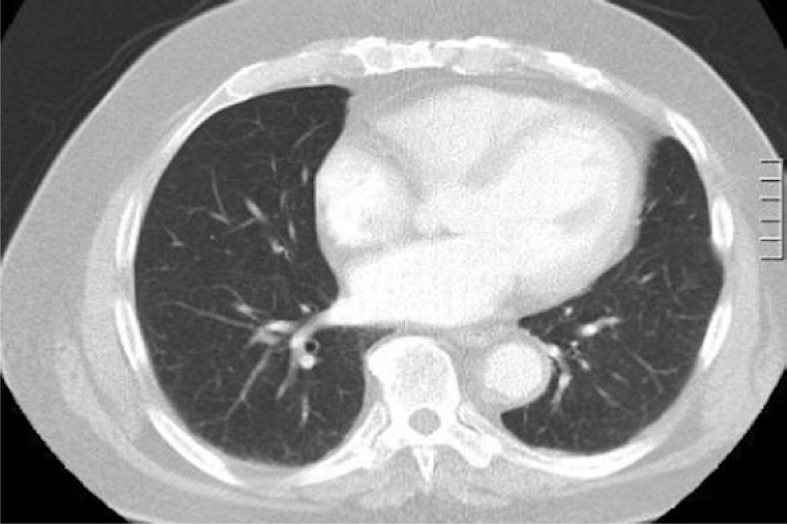
Representative image from initial staging CT; minor emphysematous changes

A 74-year-old man with a history of metastatic colorectal cancer was admitted with a four week history of progressive dyspnoea and haemoptysis. He had undergone a right hemicolectomy three and a half years previously for a Duke’s A colorectal cancer. Three years post resection, a large single liver metastasis was identified with no other evidence of metastatic disease on PET scanning. CT examination of the chest at the time demonstrated only mild emphysematous changes (Figure 1).

**FIGURE 2.**
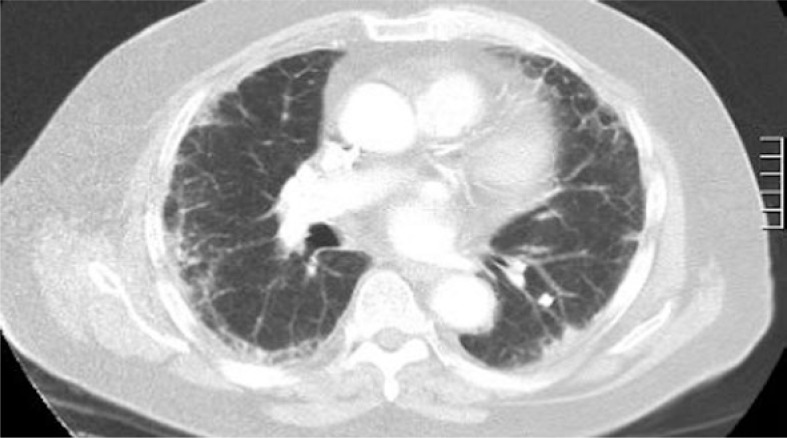
Representative image from re-staging CT January 2009; bilateral peripheral subpleural reticular changes and interlobular septal thickening without evidence of lung metastases.

The patient was commenced on FOLFOX chemotherapy (oxaliplatin, 5-fluorouracil and leucovorin) and underwent six cycles from October to December 2008. A re-staging CT scan in January 2009 demonstrated progressive malignant disease with multiple new metastases within the liver. Chest imaging at this time revealed bilateral peripheral subpleural reticular changes and interlobular septal thickening without evidence of overt lung metastases (Figure 2). No changes to treatment occurred and the patient was planned for second line systemic chemotherapy.

**FIGURE 3.**
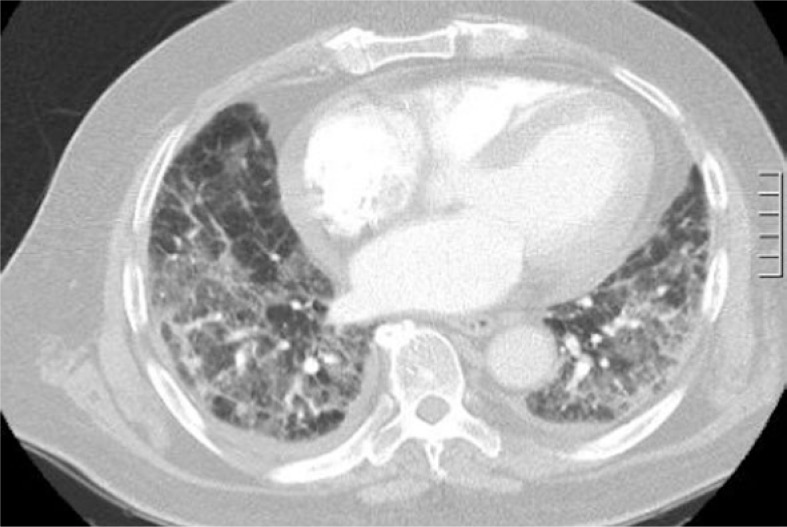
Representative image from CT pulmonary angiogram February 2009; diffuse infiltrates with interlobular septal thickening.

The patient subsequently presented in early February 2009 with four weeks of worsening dyspnoea and haemoptysis. His Creactive protein level was moderately elevated although white cell count and differential were normal. Arterial blood gases demonstrated severe type I respiratory failure. CT pulmonary angiogram excluded pulmonary embolism but demonstrated new patchy infiltrates with interlobular septal thickening (Figure 3). Sputum samples were unable to be obtained and the patient was considered to be too high risk to undergo bronchoscopy.

He was commenced on empirical broad spectrum antibiotics and high dose intravenous corticosteroid therapy. His respiratory failure worsened and after discussion with both the patient and his family, mechanical ventilation was not administered. The patient died four days after admission from progressive respiratory failure.

Post-mortem examination did not demonstrate evidence of an infective aetiology on staining and culture of fresh specimens. There was also no evidence of emboli, thrombus or malignant infiltration within the lungs. Histological examination demonstrated widespread generalised changes within both lungs, with oedema, intra-alveolar haemorrhage and fibrin deposition along with interstitial and intraluminal fibroblastic proliferation without mature fibrosis. These findings were consistent with diffuse alveolar damage.

## Discussion

Diffuse alveolar damage (DAD) is a common histopathological finding in patients with ARDS and is commonly associated with infections (both respiratory and systemic). It is more frequently seen in immunocompromised patients. Although drug reactions are a relatively uncommon cause of DAD, chemotherapeutic agents are overrepresented as potential precipitants. Mortality following histologically proven DAD obtained from surgical lung biopsy has been reported to be greater than 50%.^7^

Oxaliplatin, in combination with 5-fluorouracil and leucovorin (FOLFOX) has been shown to be an effective regimen for the treatment of advanced colorectal cancer.^1^,^10^–^12^ Goldberg *et al.* demonstrated improved response rate, median time to progression and overall survival in patients with advanced colorectal cancer treated with FOLFOX in comparison to a regimen utilising Irinotecan instead of Oxaliplatin.^1^ The most frequently reported side effects in this and other studies utilising FOLFOX have been cytopenias, peripheral neuropathy and diarrhoea.^1^,^10^ Pulmonary toxicity associated with this regimen has been infrequently described in a small number of case reports.^4^–^8^,^13^–^15^ In cases where a tissue diagnosis was obtained, DAD was the most frequent histopathological pattern obtained, although organising pneumonia has also been described.^16^ Authors have suggested apparent clinical benefit with prompt cessation of FOLFOX therapy and the use of high dose systemic corticosteroids.^8^,^14^,^17^

Although pulmonary toxicity from FOLFOX appears to be an uncommon and apparently idiosyncratic effect of therapy, this case highlights the importance of clinicians being aware of this potentially fatal complication and the importance of regular follow-up.^18^ Early changes consistent with acute interstitial lung disease were present on a routine re-staging CT chest over four weeks prior to this patient’s acute presentation and it is possible that recognition of this problem may have altered the outcome.

## Conclusions

Given the temporal relationship between the use of FOLFOX and the onset of radiological changes and clinical symptoms, we believe that FOLFOX is the likely precipitant in this case. Whilst this appears to be an uncommon adverse effect, early recognition may allow prompt cessation of FOLFOX therapy and initiation of systemic corticosteroids, and this has the potential to improve outcomes for such patients.
